# U.K. National Radiological Protection Board Radon Calibration Procedures

**DOI:** 10.6028/jres.095.014

**Published:** 1990

**Authors:** K. D. Cliff

**Affiliations:** National Radiological Protection Board, Chilton, Didcot, Oxon, OX11 ORQ, U.K.

**Keywords:** aerosol generation, alphaspectrometry, calibration chamber, radon daughter measurement, radon gas measurement, statistical uncertainty

## Abstract

A procedure for the calibration of instruments for the detection of ^222^Rn in air is described. The method is based on the alpha-spectrometric determination of the concentration in air of ^218^Po in the calibration chamber. The calibration chamber is described, together with the method of maintaining a high aerosol concentration. The ^218^Po concentration at steady state in the chamber is found to be 98% of the ^222^Rn concentration typically. An assessment of the sources of uncertainty in the method presented indicate that the ^222^Rn concentration in the chamber can be determined with an overall uncertainty of about 7% at the 95% confidence level.

## 1. The Calibration Chamber

The calibration of radon (^222^Rn) equipment at the U.K. National Radiological Protection Board (NRPB) is carried out in a sealed chamber of 43 m^3^ volume. The chamber has a double door (air-lock) allowing entry and exit with the minimum disturbance of the contained atmosphere. The ventilation rate of the chamber can be controlled by a variable speed exhaust fan and adjustable inlet apertures. When used for the calibration of instruments for the detection of radon, however, the exhaust fan is removed and all leakage apertures sealed. Under these conditions, the inherent infiltration is 0.006 to 0.008 air changes per hour as determined by the release of nitrous oxide and by monitoring its concentration as a function of time with a Miran-101^1^ infra-red spectrophotometer (Fox Wilboro Inc., Norwalk, Connecticut).

Connected to the chamber is a temperature-controlled tube furnace in which carnauba wax is heated, while air from the chamber is circulated through the furnace and returned to the chamber. The furnace is operated at a temperature of 200 °C with a flow rate over the wax of 3 L min^−1^, while a flow rate of 0.15 L min^−1^ is maintained through the annulus surrounding the boat containing the wax. The resulting aerosol has a nominal count median diameter of 0.1 *µ*m with geometric standard deviation of 1.6. At the steady state this aerosol generator maintains an aerosol concentration in the chamber of 3 × 10^4^ particles cm^−3^.

Radon is generated in the chamber by bubbling air through solutions of radium-226 chloride. These liquid radon sources alone provide a steady-state radon concentration of 2500 Bq m^−3^; with the addition of dry radon sources, about 8000 Bq m^−3^ is attained.

## 2. Radon Daughter Measurement

Measurement of the radon concentration in the chamber is principally by alpha-spectrometric determination of the airborne radon daughters by the method proposed by Nazaroff et al. [1]. The decay scheme for radon is shown in table 1, together with that for ^220^Rn (thoron). A sample of the air in the chamber is drawn through a membrane filter (type AA, 0.8 *µ*m pore diameter, Millipore U.K. Ltd., Watford) at a nominal flow rate of 20 L min^−1^ for a period of 10 min by means of a high-vacuum pump (type EB3A, Edwards, Bristol). The total volume sampled is measured with a dry gas meter (type G4 Mk2sl, IGI Meters Ltd., London) attached to the exhaust of the pump. The gas meter reading is corrected for the temperature of the exhaust air; the systematic uncertainty in the volume measured has a semi-range of 2%. Repeated measurements with a number of clean filters has shown that the flow rate does not vary over a 10 min sampling period by more than 0.2% of the mean value. Any change in flow rate occurs during the first 3 min of the sampling period; thereafter it is constant. The control of all timing periods is effected by a personal computer (PC) as is the analysis of the measured spectra; timing errors are negligible. The membrane filter is transferred to the counting system during a 1 min interval between the end of the sampling period and the start of the first spectrum acquisition. The counting system comprises a drawer counter, to hold the filter, with a 450 mm^2^ silicon surface barrier detector (type A-025-450-100, EG&G Ortec, Oak Ridge, Tennessee), a preamplifier (type 124, EG&G Ortec), a spectrometry amplifier (type 451, EG&G Ortec), and a multi-channel analyzer (MCA) (type 7500, EG&G Ortec).

As soon as the sample filter is in position beneath the detector, the space between the filter and the detector is partially evacuated to 8.7 × 10^4^ Pa below atmospheric pressure by a small pump (type XX55, Millipore U.K. Ltd., Watford). When the 1 min interval following the end of the sampling period has elapsed, an alpha particle energy spectrum is accumulated for 10 min. A delay of 10 min follows before a second spectrum is accumulated over a period of 19 min. At the end of each spectrum accumulation period, the integral counts from the channels of the MCA corresponding to alpha particle energy ranges of 1.0 to 6.15 MeV, 6.15 to 8.0 MeV, and 8.0 to 9.8 MeV are acquired by the PC. These energy ranges encompass the peaks from ^218^Po (6.00 MeV), ^214^Po (7.69 MeV), and ^212^Po (8.78 MeV). The data are corrected for the fraction of counts in the ^218^Po channels due to alpha particles from ^214^Po that are degraded in energy in their passage from the site of decay to the detector. Similarly, corrections are applied for the fractions of alpha particles from ^212^Po that occur in the ^214^Po channels and from ^212^Bi that occur in the ^218^Po channels; these last corrections are necessary as the chamber is used occasionally for the measurement of ^220^Rn daughters. Finally, the volume of air sampled and the alpha particle detection efficiency are input to the PC, and the results are calculated to give the activity concentrations of ^218^Po, ^214^Pb, ^214^Bi/^214^Po, and potential alpha energy concentration (PAEC).

The detection efficiency for the spectrometry system has been determined by comparison with a laboratory standard alpha counter, the efficiency of which was established with a reference ^241^Am source (Amersham International Ltd., Amersham). The alpha particle emission rate for this reference source is known with a semi-range uncertainty of 2%. A filter sample of ^220^Rn daughters that had been taken some 16 h earlier was counted alternately on the spectrometry system and the laboratory alpha counter; this was repeated several times, corrections being made for the decay of ^212^Pb with a half-life of 10.6 h. The efficiency of the spectrometry system was also determined using the reference ^241^Am source directly with an appropriate correction for source-to-detector geometry. The efficiency found by the two methods differed by less than 1%.

Radon calibrations are always carried out under virtual steady state conditions. These conditions are achieved if the chamber has been undisturbed for at least 20 d (beyond 20 d a constant radon concentration exists in the chamber) or by removing all radon sources at least 3 h prior to the calibration (beyond 3 h, the state of equilibrium between radon and its short-lived daughters is constant and all decay is determined by the half-life of radon). A radon concentration in the chamber of not less than 2000 Bq m^−3^ is also used. Under these conditions, the uncertainty in the measurement of the ^218^Po activity concentration has a standard deviation arising from counting statistics alone of less than 2%.

## 3. Radon Gas Related to ^218^Po

The state of equilibrium between radon gas and radon daughters in the sealed chamber was calculated using the room model devised by Jacob [2] and refined by Porstendorfer et al. [3]. Repeated measurements of radon daughter concentrations in the chamber using the spectrometry system when the ^218^Po concentration was assessed to be in the range 2000–2400 Bq m^−3^ yielded mean activity ratios ^218^Po : ^214^Pb : ^214^Bi of 1 : 0.960 :0.925. These data were fitted to the room model under the assumption that the deposition (plate-out) constant for radon daughters attached to the aerosol was two orders of magnitude smaller than that for unattached radon daughters. The model is quite insensitive to the value of the deposition rate for attached radon daughters: for example, changing the ratio between the deposition rate constant for attached and unattached daughters from 0.01 to 0.1 typically changes the predicted ratio of total ^218^Po activity to that of radon gas by less than 11%. The probability that a ^214^Pb atom formed by the decay of ^218^Po attached to an aerosol particle will be detached by recoil [4] is taken as 0.83. Table 2 gives the range of parameter values for the room model that provide an adequate fit to the data for the chamber. The range of the ratio total ^218^Po to radon is from 0.967 to 0.990. To account for modelling uncertainties, this ratio is taken to be 0.98 with a semi-range systematic uncertainty of 2%.

The range of the rate constant for attachment of radon daughters to aerosols implies, for an aerosol concentration of 3 × 10^4^ particles cm^−3^ a range in average attachment rate coefficient from 2.7 × 10^−3^ to 6.3 × 10^−3^ cm^3^h^−1^. The attachment rate coefficient is the coefficient of proportionality relating the aerosol concentration to the rate constant for the attachment of radon daughters to aerosols. Reported values for this coefficient [5] vary from 1.8 × 10^−3^ to 6.8 × 10^−3^ cm^3^ h^−1^, and the values calculated here lie comfortably within this interval. The range of values found for the deposition rate constant for unattached radon daughters is rather lower than the range of 10 to 40 h^−1^ considered typical [6] in dwellings. Air movement within the chamber is low, however, and most surfaces are smooth and electrically conducting. The surface area to volume ratio for the chamber is about 2.8 m^−1^. The corresponding deposition velocities for unattached radon daughters required to fit the data are in the range 1.07 to 1.79 m h^−1^, similar to those found by Knutson et al. of 1.19 to 2.38 m h^−1^ in another metal exposure chamber [7]. It is concluded that the derived parameters for the room model are good and that the radon concentration may be taken to be 102% of the concentration of ^218^Po in the chamber as measured by the spectrometry system, with a semi-range systematic uncertainty of 2%.

## 4. Summary of Uncertainties

All sources of uncertainty in the determination of radon concentration by the spectrometric measurement of ^218^Po concentration are shown in table 3. There are unaccounted uncertainties, such as linearity of the spectrometry system, and it has been estimated that these produce a maximum standard deviation of 2%. The overall systematic uncertainty, as a standard deviation, is found [8] by adding the individual semi-ranges in quadrature and dividing the result by the square root of 3. This is derived by considering each systematic uncertainty to have a rectangular distribution, i.e., all values over the range of uncertainty are deemed to be equally likely. The overall random uncertainty is obtained by the addition of each uncertainty in quadrature. The final overall uncertainty is simply the sum in quadrature of the systematic and random uncertainties derived for the same confidence level.

It is concluded that determination of radon concentration at NRPB by the alpha spectrometric measurement of the concentration of ^'^Po provides a result that has an uncertainty of around 7% at the 95% confidence level.

## Figures and Tables

**Table 1:** Decay schemes for ^222^Rn (radon) and ^220^Rn (thoron), short-lived daughters only

Nuclide	Half-life	Mode of decay	*α*-Energy	MeV
^222^Rn	3.823 d	*α*	5.48	
^218^Po	3.11 min	*α*	6.00	
^214^Pb	26.8 min	*β,γ*		
^214^Bi	19.7 min	*β,γ*		
^214^Po^a^	164 µs	*α*	7.69	

^220^Rn	55 s	*α*	6.28	
^216^Po	0.16 s	*α*	6.78	
^212^Pb	10.6 h	*β,γ*		
^212^Bi	60.5 min	*α,β,γ*	6.94	25%
			6.08	10%
			other	1%
^212^Po	0.3 µs	*α*	8.78	64%

a ^214^Po is always in equilibrium with ^214^Bi.

**Table 2:** Range of parameter values that account for the radon daughter conditions in the exposure chamber

Detachment probability for ^214^Pb on formation	0.83
Ratio of deposition rate constants for attached and unattached radon daughters	0.01
Ventilation rate	0.006–0.008 h^−1^
Rate constant for attachment of radon daughters to aerosols	80–190 h^−1^
Deposition rate constant for unattached radon daughters	3–5 h^−1^
Ratio of activity concentrations of ^218^Po to radon	0.967–0.990
*Assumed* mean activity ratio, ^218^Po to radon	0.98
*Assumed* range of activity ratio, ^218^Po to radon	0.96–1.00

**Table 3:** Assessed uncertainties in the measurement of radon activity concentration

Systematic uncertainties	Per cent	
Flow rate:	Semi-range	2
Reference ^241^Am source:	Semi-range	2
Modelling:	Semi-range	2
Overall systematic, 95% confidence interval:		±3.9%

Random uncertainties	Standard deviation %
Counting statistics, determination of ^218^Po:	2
Counting statistics, efficiency calibration:	1
Overall random, 95% confidence interval:	±4.48%
Estimated unaccounted uncertainty, 95% confidence interval	±3.96%
95% interval overall:	±7.1%

## Footnotes

1 Certain commercial equipment, instruments, or materials are identified in this paper to specify adequately the experimental procedure. Such identification does not imply recommendation or endorsement by the National Institute of Standards and Technology, nor does it imply that the materials or equipment identified are necessarily the best available for the purpose.
